# Supplementary material for: A conserved membrane protein negatively regulates Mce1 complexes in mycobacteria
Source: Nat Commun. 2023 Sep 22;14:5897. doi: 10.1038/s41467-023-41578-y (PMC10517005; doi:10.1038/s41467-023-41578-y)

Supplementary information for

A conserved membrane protein negatively regulates Mce1 complexes in mycobacteria

Authors: Yushu Chen, Yuchun Wang, Shu-Sin Chng*

*Corresponding author. Email: chmchngs@nus.edu.sg

This PDF file includes:

Supplementary Figures 1 to 8
Supplementary Tables 1 to 5
Supplementary References 1 to 13
Uncropped gels and blots for Supplementary Figure 6

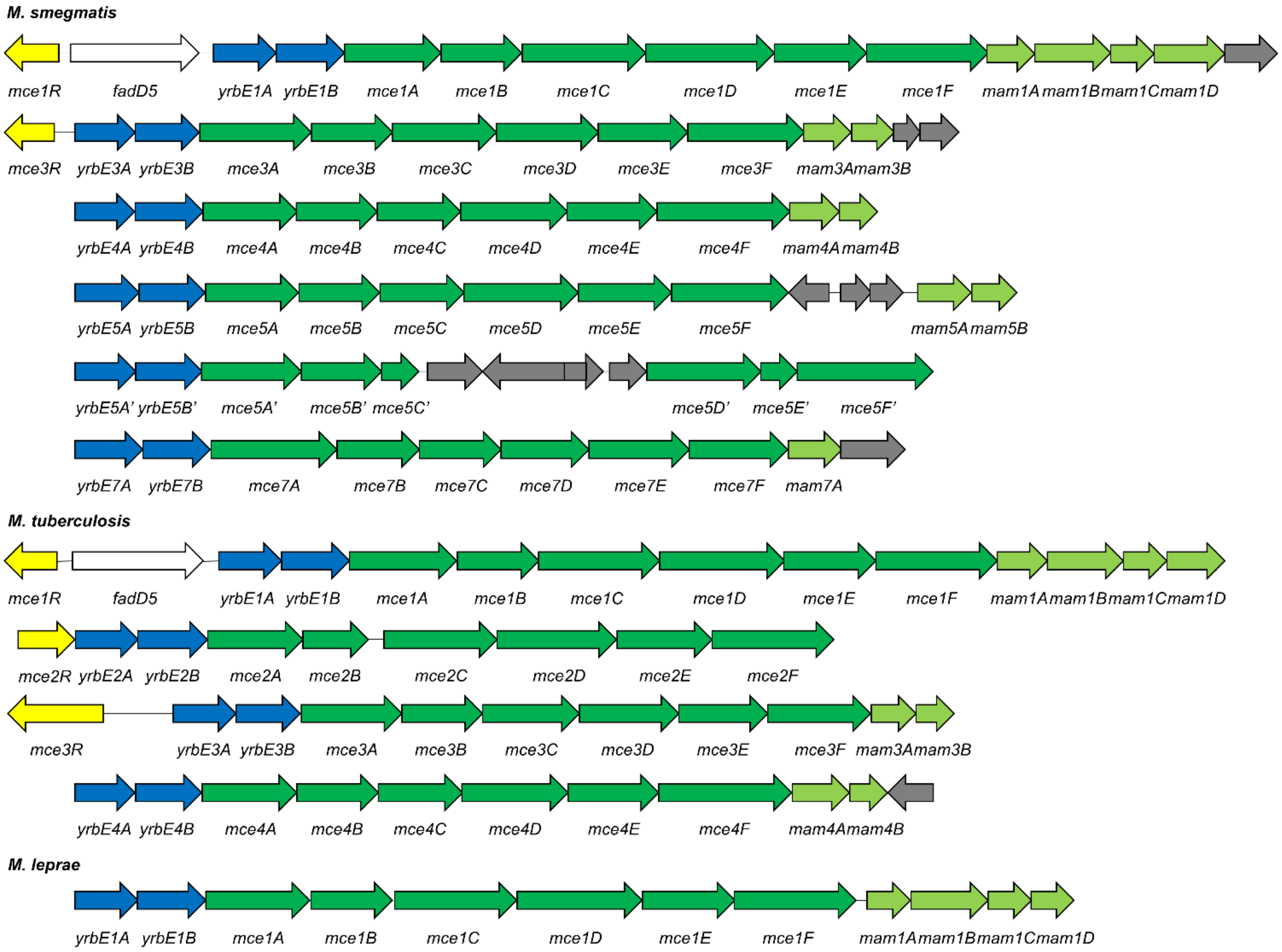

Supplementary Figure 1. A schematic representation of the *mce* genetic loci in *M. smegmatis*, *M. tuberculosis* and *M. leprae*. Each straight arrow represents an individual gene. The lengths of the arrows are scaled to the lengths of the genes. The adjacent gene that encodes the transcriptional regulator of some operons is colored in yellow. *yrbE* genes are colored in blue, *mce* genes dark green, *mam* genes light green, and unrelated genes grey. *mce1* operons in *M. smegmatis* and *M. tuberculosis* contain an additional gene encoding a putative fatty acyl-CoA synthase (*fadD5*, white). The *mce5* operon contains an insertion of unrelated genes between *mce5* and *mam5* genes. The *mce5bis* operon (*mce5'*) contains an insertion between *mce5C'* and *mce5D'*.

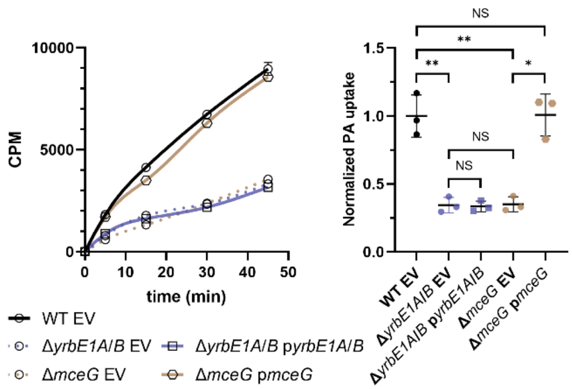

Supplementary Figure 2. The Δ*mceG* strain can be complemented by exogenous expression of *mceG*. [^{14}C]-palmitic acid uptake profiles and rates of indicated *M. smegmatis* strains. The uptake profile (left) shows accumulated radioactivity counts in cells over time, and is representative of at least three independent experiments. Each data point (mean ± standard deviation) represents results from three technical replicates. CPM, count per minute. Uptake rates (right) are quantified based on [^{14}C]-palmitic acid levels after 30 min incubation. The uptake of individual strains is normalized to that of WT cells harboring the empty vector (WT EV). Mean ± standard deviation of three biological replicates is shown for each group. EV, empty vector (pJEB402). One-way repeated measures ANOVA: NS, not significant; ** $p < 0.01$. WT EV vs Δ*yrbE1A/B* EV ($p = 0.0074$), WT EV vs Δ*mceG* EV ($p = 0.008$), Δ*yrbE1A/B* EV vs Δ*mceG* EV ($p = 0.24$), Δ*yrbE1A/B* EV vs Δ*yrbE1A/B* p*yrbE1A/B* ($p = 0.47$), WT EV vs Δ*mceG* p*mceG* ($p = 0.91$), Δ*mceG* EV vs Δ*mceG* p*mceG* ($p = 0.012$). The Δ*yrbE1A/B* strain could not be complemented by expressing *yrbE1A/B*, very likely due to polar effects on downstream genes. Nevertheless, both the Δ*yrbE1A/B* and Δ*mceG* strains represent cells that have lost Mce1 function completely, therefore useful as negative controls.

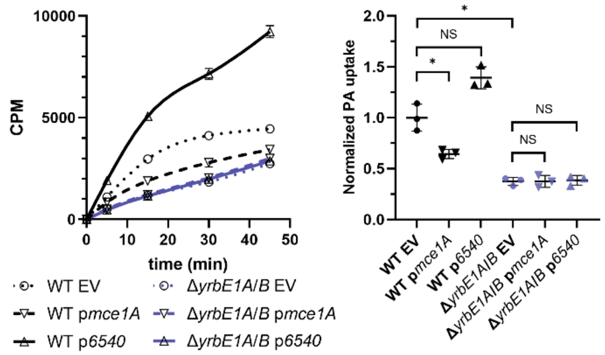

Supplementary Figure 3. Mce1A and MSMEG_6540 may compete to form distinct Mce1 complexes. [^{14}C]-palmitic acid uptake profiles and rates of indicated *M. smegmatis* strains. The uptake profile (left) shows accumulated radioactivity counts in cells over time, and is representative of at least three independent experiments. Each data point (mean ± standard deviation) represents results from three technical replicates. CPM, count per minute. Uptake rates (right) are quantified based on [^{14}C]-palmitic acid levels after 30 min incubation. The uptake of individual strains is normalized to that of WT cells harboring the empty vector (WT EV). Mean ± standard deviation of three biological replicates is shown for each group. EV, empty vector (pJEB402); *6540*, *MSMEG_6540*. One-way repeated measures ANOVA: NS, not significant; * $p < 0.05$. WT EV vs WT p*mce1A* ($p = 0.021$), WT EV vs WT p*6540* ($p = 0.099$), WT EV vs Δ*yrbE1A/B* EV ($p = 0.014$), Δ*yrbE1A/B* EV vs Δ*yrbE1A/B* p*mce1A* ($p = 0.96$), Δ*yrbE1A/B* EV vs Δ*yrbE1A/B* p*6540* ($p = 0.36$).

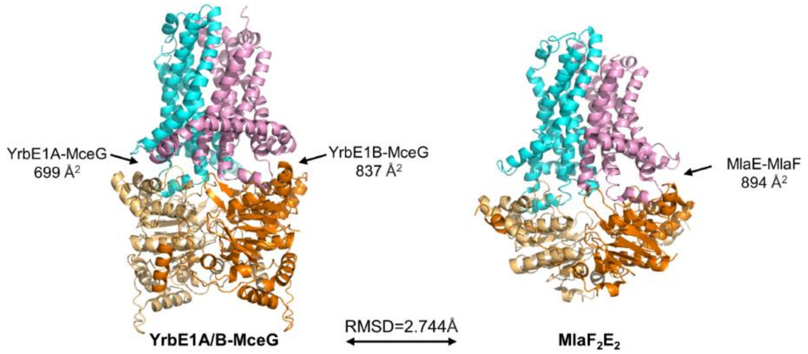

Supplementary Figure 4. Comparison of the AlphaFold2 structural model of YrbE1A/B-MceG and cryo-electron microscopy structure of MlaFE (PDB: 7CH6, with MlaB removed)[1]. Left panel, cyan: YrbE1A, pink: YrbE1B, light orange and orange: MceG; right panel, cyan and pink: MlaE, light orange and orange: MlaF. Calculated interface areas are indicated.

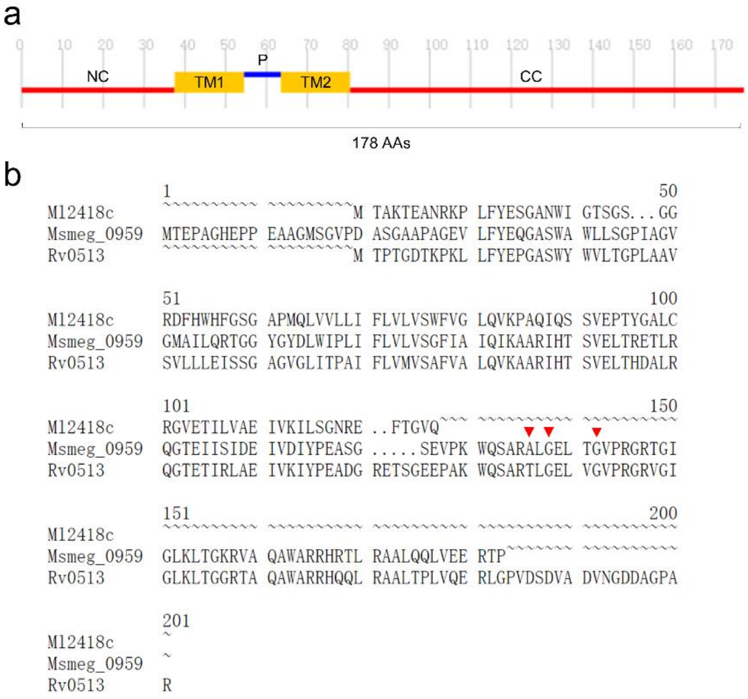

Supplementary Figure 5. MSMEG_0959 is a transmembrane protein conserved in mycobacteria. a A predicted topology model of MSMEG_0959. The length of MSMEG_0959 is 178 amino acids (AAs). Cytoplasmic domains (N-terminal and C-terminal, NC and CC) are colored in red, the periplasmic (P) loop in blue, and the transmembrane (TM) domains in yellow. **b** Sequence alignment of MSMEG_0959 and its homologs in *M. tuberculosis* (Rv0513) and *M. leprae* (ML2418). The protein encoded by *ML2418* lacks the sequence corresponding to a large portion of the C-terminal cytoplasmic domain in MSMEG_0959 due to an early stop codon (only 103 amino acids compared to 178 for MSMEG_0959). The red arrowheads indicate residues that were mutated to prolines on the MSMEG_0959 variant (MSMEG_0959$_{AGG3P}$) that no longer competes with MceG for binding to YrbE1B.

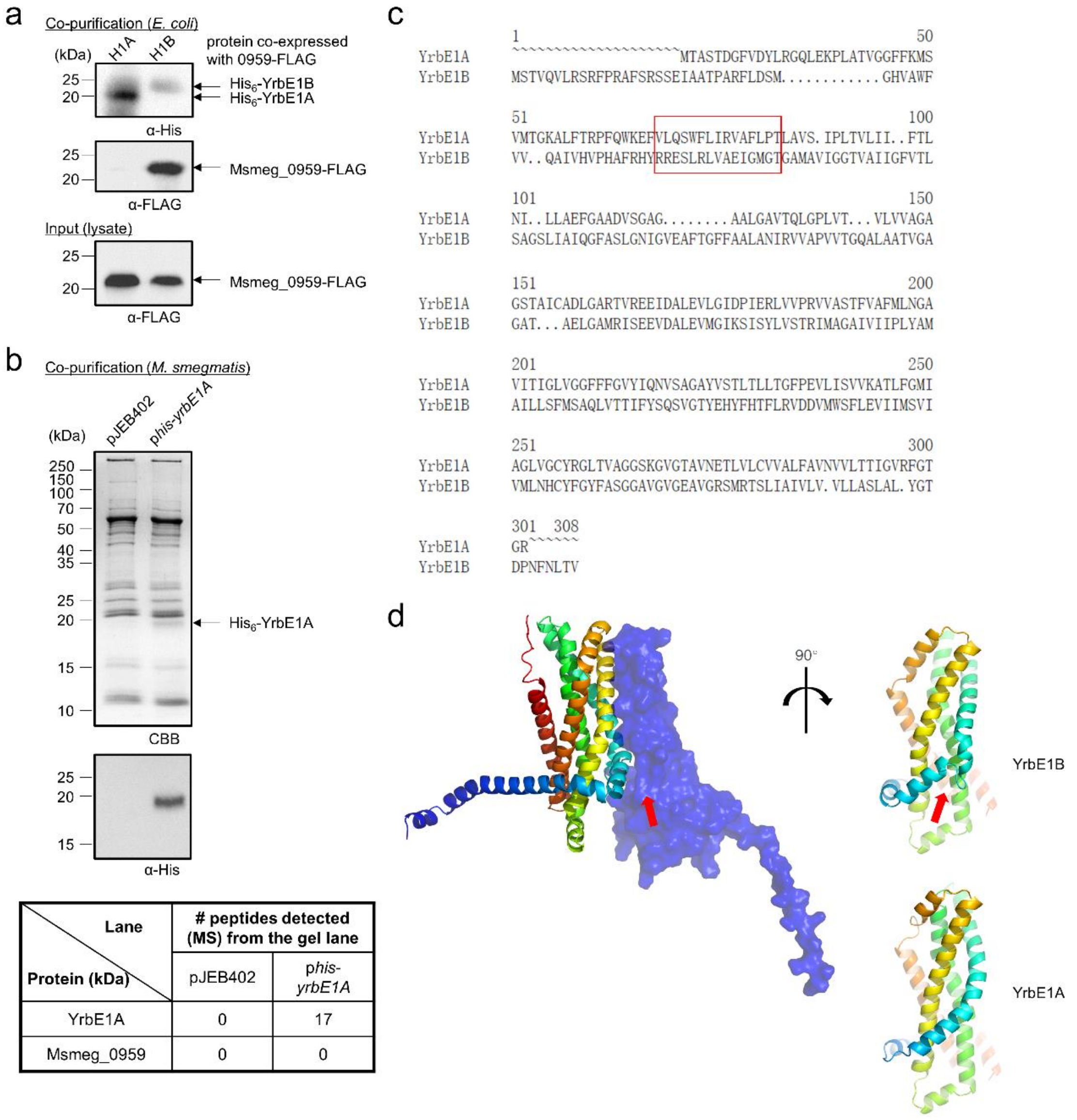

Supplementary Figure 6. MSMEG_0959 co-purifies with YrbE1B but not YrbE1A. a SDS-PAGE and α-His/FLAG immunoblot analyses of proteins affinity-purified from *E. coli* cells expressing His-tagged YrbE1A (H1A) or YrbE1B (H1B) together with FLAG-tagged MSMEG_0959. **b** SDS-PAGE and α-His immunoblot analyses of proteins affinity-purified from *M. smegmatis* cells expressing His-tagged YrbE1A (p*his-yrbE1A)*. pJEB402 was used as the empty vector control. The two entire gel lanes were subjected to MS/MS protein identification. The table shows total numbers of peptides detected for YrbE1A and MSMEG_0959. CBB, Coomassie brilliant blue. **c** Sequence alignment of YrbE1A and YrbE1B. The region of YrbE1B for the putative helix that is required for the interaction of YrbE1B with MSMEG_0959 and the corresponding region of YrbE1A are boxed in red. **d** Left panel: An AlphaFold2 model of YrbE1B (rainbow) -MSMEG_0959 (shown as blue surface). Right panel: comparison of the putative regions facing MSMEG_0959 in YrbE1B and YrbE1A. The red arrows point towards the short helix and its adjoining loop on YrbE1B that likely determines its interaction with MSMEG_0959, which is absent at the same region of YrbE1A. **a** and **b** (other than the MS/MS analysis) were repeated three times independently with similar results.

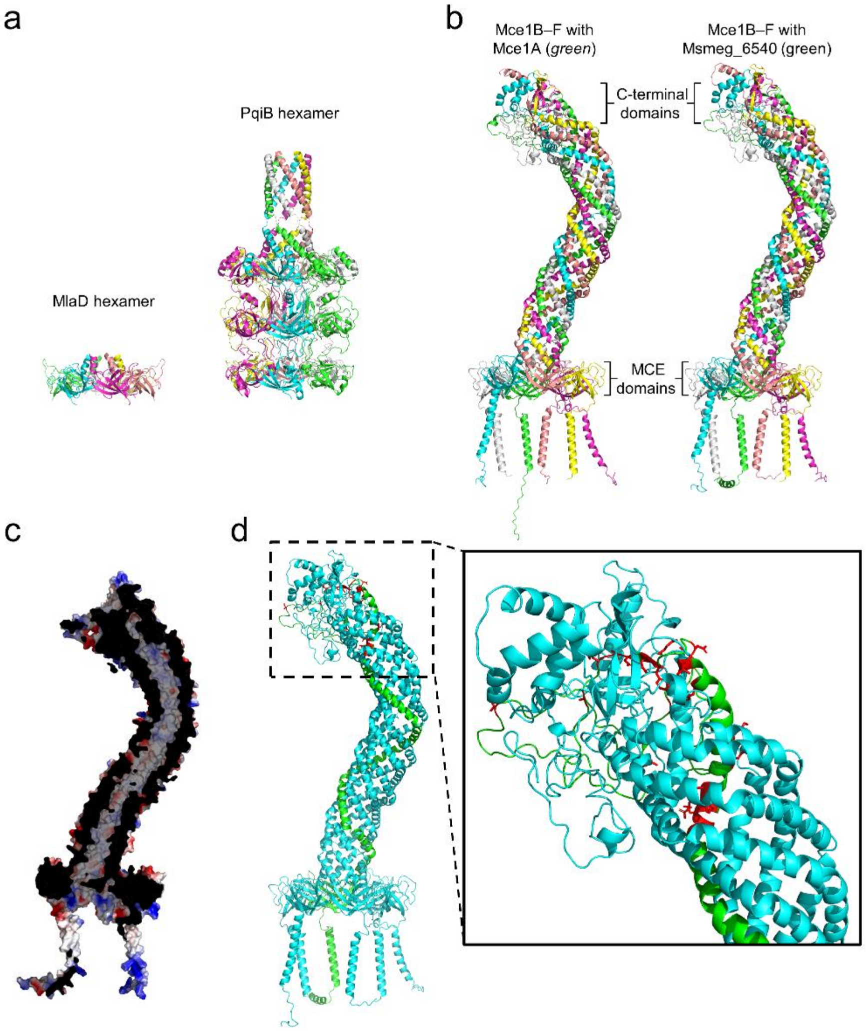

Supplementary Figure 7. Mce1B–F form putative heterohexamers with Mce1A or MSMEG_6540. a Structures of the hexamer of MlaD periplasmic domains (left, single MCE domain per protomer, PDB: 5UW2) and the hexamer of PqiB periplasmic domains (right, three MCE domains per protomer, PDB: 5UVN)[2]. **b** AlphaFold2 structural models of putative heterohexamers formed by either Mce1A or MSMEG_6540 with Mce1B–F. Long unstructured C-terminal tails of Mce1C, Mce1D and Mce1F in the models were removed for the purpose of clarity. green: Mce1A or MSMEG_6540, yellow: Mce1B, salmon: Mce1C, cyan: Mce1D, magenta: Mce1E, grey: Mce1F. **c** Cross-section view of the electrostatic surface map of the hexamer formed by MSMEG_6540 and Mce1B–F, revealing a hydrophobic tunnel spanning the entire assembly. **d** The AlphaFold2 structural model of the hexamer formed by MSMEG_6540 (green) and Mce1B–F (cyan) with residues in the C-terminal domain of MSMEG_6540 differing from those on Mce1A highlighted in red.

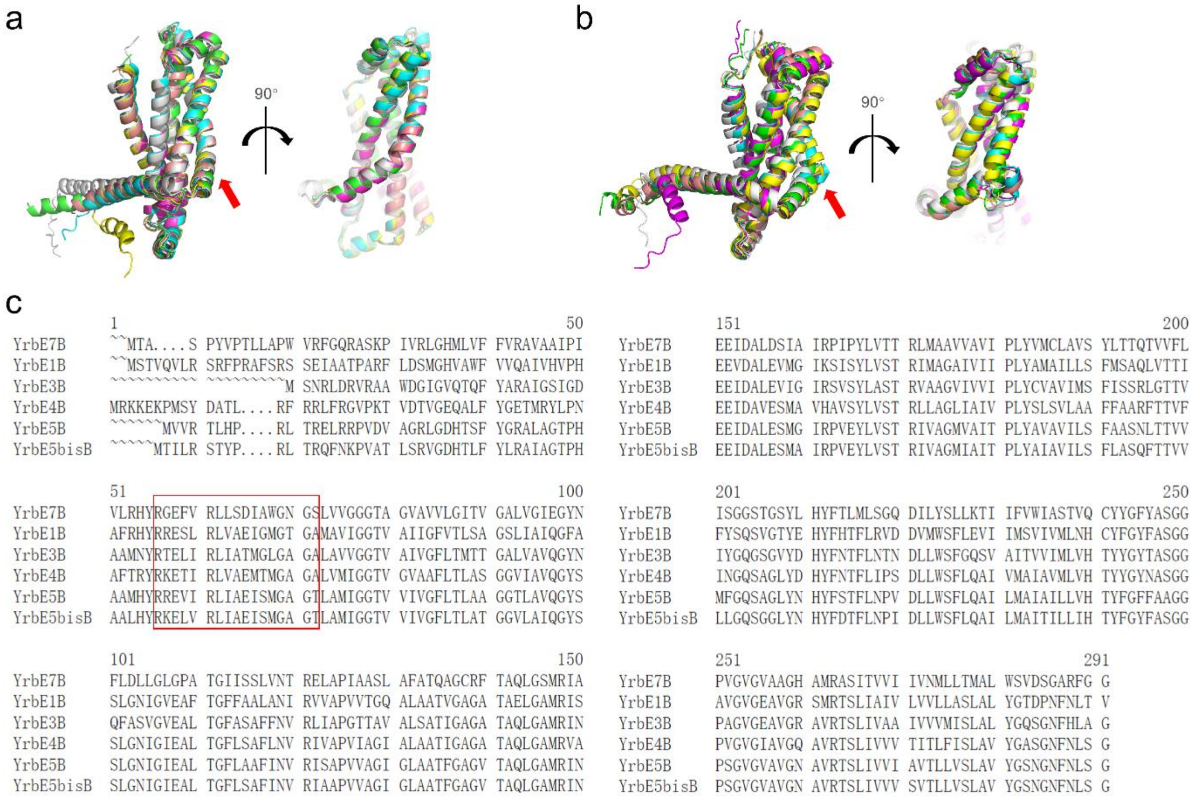

Supplementary Figure 8. The putative MSMEG_0959 binding motif may be conserved across YrbEBs. **a** Overlay of Alphafold2 structural models of six YrbEAs in *M. smegmatis*. **b** Overlay of Alphafold2 structural models of six YrbEBs in *M. smegmatis*. In **a** and **b**, the region where YrbE1B is predicted to interact with MSMEG_0959, and the corresponding region in YrbEAs, are indicated with red arrows. **c** Sequence alignment of six YrbEBs in *M. smegmatis*. The putative helix that is required for the interaction of YrbE1B with MSMEG_0959 is boxed in red.

Supplementary Table 1. Percentage sequence identity of Mce1A and its homologs with other Mce proteins in *M. smegmatis*.

% seq identity	Mce1A	6540	5818	Mce1B	Mce1C	Mce1D	Mce1E	Mce1F	Mce3A	Mce4A	Mce5A	Mce5b isA	Mce7A
Mce1A		79.8	59.8	14.9	16.3	18.4	20.0	14.7	28.1	28.7	31.9	29.6	16.3
Msmeg_6540			57.7	16.7	17.1	14.5	19.6	18.7	26.1	26.8	26.9	28.2	17.9
Msmeg_5818				17.8	16.4	13.5	22.0	19.1	28.2	28.5	30.8	30.3	18.9

Supplementary Table 2. Area of interfaces between each pair of TMD and NBD protomer within different ABC transporters with heterodimeric TMDs. PDB ID: $LptB_2FG$[3], 6MHZ; $LolCD_2E$[4], 7ARM; $SugABC_2$[5], 7CAG; $MalFGK_2$[6], 2R6G; $AlgM1M2S_2$[7], 4TQU. For YrbE1A/B-MceG, the AlphaFold2 model[8,9] was used for the interface analysis.

**ABC transporter**	**YrbE1A/B-MceG**		**$LptB_2FG$**		**$LolCD_2E$**		**$SugABC_2$**		**$MalFGK_2$**		**$AlgM1M2S_2$**	
TMD-NBD	A-G	B-G	F-B	G-B	C-D	E-D	A-C	B-C	F-K	G-K	M1-S	M2-S
Interface area* (Å^2)	699	837	807	1081	1286	1022	756	1053	775	1024	836	797

* Calculated using the interface analysis function in ChimeraX 1.4.

Supplementary Table 3. *M. smegmatis* strains used in this study.

**Strains used**	**Source**
mc^2155	lab collection
mc^2155 Δ*mce1R*	this study
mc^2155 Δ*mceG*	this study
mc^2155 Δ*mce1A*	this study
mc^2155 Δ*MSMEG_6540*	this study
mc^2155 Δ*mce1B*	this study
mc^2155 Δ*mce1C*	this study
mc^2155 Δ*mce1D*	this study
mc^2155 Δ*mce1E*	this study
mc^2155 Δ*mce1F*	this study
mc^2155 Δ*yrbE1A/B*	this study
mc^2155 Δ*mce1A*Δ*MSMEG_6540*	this study

Supplementary Table 4. Plasmids used in this study for protein expression and gene deletion in *M. smegmatis*.

plasmid	description	source
pJEB402	an integrative vector containing an *attP* site; express genes under an MOP promoter; Kan^R	Lee & Hatfull[10]
pJEB402*his$_6$-yrbE1A*	to express YrbE1A with an N-terminal His-tag under an MOP promoter; Kan^R	this study
pJEB402*yrbE1A/B*	to express YrbE1A/B under an MOP promoter; Kan^R	this study
pJEB402*mceG*	to express MceG under an MOP promoter; Kan^R	this study
pJEB402*mce1A*	to express Mce1A under an MOP promoter; Kan^R	this study
pJEB402*MSMEG_6540*	to express MSMEG_6540 under an MOP promoter; Kan^R	this study
pJEB402*mce1B*	to express Mce1B under an MOP promoter; Kan^R	this study
pJEB402*mce1C–F-mam1A–D*	to express Mce1C–F and Mam1A–D under an MOP promoter; Kan^R	this study
pJEB402*mce1B–F-mam1A–D*	to express Mce1B–F and Mam1A–D under an MOP promoter; Kan^R	this study
pJEB402*mce1C*	to express Mce1C under an MOP promoter; Kan^R	this study
pJEB402*mce1D*	to express Mce1D under an MOP promoter; Kan^R	this study
pJEB402*mce1E*	to express Mce1E under an MOP promoter; Kan^R	this study
pJEB402*mce1E–F-mam1A–D*	to express Mce1E–F and Mam1A–D under an MOP promoter; Kan^R	this study
pJEB402*mce1F*	to express Mce1F under an MOP promoter; Kan^R	this study
pJEB402*mam1A–D*	to express Mam1A–D under an MOP promoter; Kan^R	this study
pJEB402*mce1F-mam1A–D*	to express Mce1F and Mam1A–D under an MOP promoter; Kan^R	this study
pMV306hsp	an integrative vector containing an *attP* site; express genes under an *hsp60* promoter; Kan^R	Andrew et al.[11]
pMV306hsp*his$_6$-yrbE1A*	to express YrbE1A with an N-terminal His-tag under an *hsp60* promoter; Kan^R	this study
pMV306hsp*his$_6$-yrbE1B*	to express YrbE1B with an N-terminal His-tag under an *hsp60* promoter; Kan^R	this study
pMV306hsp*MSMEG_0959$_{AGG3P}$*	to express MSMEG_0959$_{AGG3P}$ under an *hsp60* promoter; Kan^R	this study
pMV306hsp*MSMEG_0959$_{AGG3P}$-FLAG+his$_6$-yrbE1B*	to express MSMEG_0959$_{AGG3P}$ with a C-terminal FLAG-tag and YrbE1B with an N-terminal His-tag under an *hsp60* promoter; Kan^R	this study

pMV306hsp*Rv0513*	to express Rv0513 under an *hsp60* promoter; Kan^R	this study
pMV306hsp*Rv0513-FLAG +his*$_6$*-yrbE1B*	to express Rv0513 with a C-terminal FLAG-tag and YrbE1B with an N-terminal His-tag under an *hsp60* promoter; Kan^R	this study
pMV306hsp*MSMEG_0959*	to express MSMEG_0959 under an *hsp60* promoter; Kan^R	this study
pMV306hsp*MSMEG_0959-FLAG+his*$_6$*-yrbE1B*	to express MSMEG_0959 with a C-terminal FLAG-tag and YrbE1B with an N-terminal His-tag under an *hsp60* promoter; Kan^R	this study
pGOAL17	a vector used as the template for the *lacZ-sacB* cassette under a P_{Ag85} promoter for screening and negative selection; Amp^R	Parish & Stoker[12]
pYUB854	a vector lacking mycobacterial replication origin; Hyg^R	Bardarov et al.[13]
pYUB854*53UTR*$_{mce1R}$*+lacZ-sacB*	to achieve unmarked deletion of *mce1R*; Hyg^R	this study
pYUB854*53UTR*$_{mceG}$*+lacZ-sacB*	to achieve unmarked deletion of *mceG*; Hyg^R	this study
pYUB854*53UTR*$_{mce1A}$*+lacZ-sacB*	to achieve unmarked deletion of *mce1A*; Hyg^R	this study
pYUB854*53UTR*$_{MSMEG\_6540}$*+lacZ-sacB*	to achieve unmarked deletion of *MSMEG_6540*; Hyg^R	this study
pYUB854*53UTR*$_{mce1B}$*+lacZ-sacB*	to achieve unmarked deletion of *mce1B*; Hyg^R	this study
pYUB854*53UTR*$_{mce1C}$*+lacZ-sacB*	to achieve unmarked deletion of *mce1C*; Hyg^R	this study
pYUB854*53UTR*$_{mce1D}$*+lacZ-sacB*	to achieve unmarked deletion of *mce1D*; Hyg^R	this study
pYUB854*53UTR*$_{mce1E}$*+lacZ-sacB*	to achieve unmarked deletion of *mce1E*; Hyg^R	this study
pYUB854*53UTR*$_{mce1F}$*+lacZ-sacB*	to achieve unmarked deletion of *mce1F*; Hyg^R	this study
pYUB854*53UTR*$_{yrbE1A/B}$*+lacZ-sacB*	to achieve unmarked deletion of *yrbE1A/B*; Hyg^R	this study

Supplementary Table 5. Plasmids used in this study for protein expression in *E. coli*.

plasmid	description	source
pET28b(+)	a high-copy vector; to express genes under an IPTG-inducible T7 promoter; pBR322 ori; Kan^R	Novagen
pET22/42	a high-copy vector; made of the backbone of pET22b(+) and the multiple cloning site of pET42a(+); to express genes under an IPTG-inducible T7 promoter; pBR322 ori; Amp^R	lab collection
pCDFDuet1	a high-copy vector; contains two multiple cloning sites to express genes under two separate IPTG-inducible T7 promoters; CloDF13 ori; $Spec^R$	Novagen
pET28b*his*$_6$-*yrbE1A*	to express YrbE1A with an N-terminal His-tag under an IPTG-inducible T7 promoter; pBR322 ori; Kan^R	this study
pET28b*his*$_6$-*yrbE1B*	to express YrbE1B with an N-terminal His-tag under an IPTG-inducible T7 promoter; pBR322 ori; Kan^R	this study
pET22/42*yrbE1A*-(*his*$_6$-*yrbE1B*)	to express YrbE1A and YrbE1B with an N-terminal His-tag using gene sequences optimized for *E. coli* codon usage under an IPTG-inducible T7 promoter; pBR322 ori; Amp^R	this study
pET22/42(*his*$_6$-*yrbE1A*)-*yrbE1B*	to express YrbE1A with an N-terminal His-tag and YrbE1B using gene sequences optimized for *E. coli* codon usage under an IPTG-inducible T7 promoter; pBR322 ori; Amp^R	this study
pET22/42*yrbE1A*-(*his*$_6$-*yrbE1B*$_{GS}$)	to express YrbE1A and YrbE1B (the helix mutant) with an N-terminal His-tag using gene sequences optimized for *E. coli* codon usage under an IPTG-inducible T7 promoter; pBR322 ori; Amp^R	this study
pCDFDuet1*mceG*	to express MceG under an IPTG-inducible T7 promoter; CloDF13 ori; $Spec^R$	this study
pCDFDuet1*mceG*$_{K43A}$	to express $MceG_{K43A}$ under an IPTG-inducible T7 promoter; CloDF13 ori; $Spec^R$	this study
pCDFDuet1*mceG-FLAG*	to express MceG with a C-terminal FLAG-tag under an IPTG-inducible T7 promoter; CloDF13 ori; $Spec^R$	this study
pCDFDuet1*MSMEG_0959-FLAG*	to express MSMEG_0959 with a C-terminal FLAG-tag under an IPTG-inducible T7 promoter; CloDF13 ori; $Spec^R$	this study
pCDFDuet1*mceG-FLAG+MSMEG_0959*	to express MceG with a C-terminal FLAG-tag and MSMEG_0959 under two separate IPTG-inducible T7 promoters; CloDF13 ori; $Spec^R$	this study
pCDFDuet1*mceG-FLAG+MSMEG_0959-FLAG*	to express MceG with a C-terminal FLAG-tag and MSMEG_0959 with a C-terminal FLAG-tag under two separate IPTG-inducible T7 promoters; CloDF13 ori; $Spec^R$	this study

pCDFDuet1*mceG-FLAG+MSMEG_0959$_{AGG3P}$-FLAG*	to express MceG with a C-terminal FLAG-tag and MSMEG_0959$_{AGG3P}$ with a C-terminal FLAG-tag under two separate IPTG-inducible T7 promoters; CloDF13 ori; Spec^{R}	this study

Supplementary References

1. Zhou, C. et al. Structural insight into phospholipid transport by the MlaFEBD complex from *P. aeruginosa*. *J. Mol. Biol.* **433**, 166986 (2021).
2. Ekiert, D. C. et al. Architectures of lipid transport systems for the bacterial outer membrane. *Cell* **169**, 273-285 (2017).
3. Li, Y., Orlando, B. J. & Liao, M. Structural basis of lipopolysaccharide extraction by the $LptB_2FGC$ complex. *Nature* **567**, 486-490 (2019).
4. Tang, X. et al. Structural basis for bacterial lipoprotein relocation by the transporter LolCDE. *Nat. Struct. Mol. Biol.* **28**, 347-355 (2021).
5. Liu, F. et al. Structural basis of trehalose recycling by the ABC transporter LpqY-SugABC. *Sci. Adv.* **6**, eabb9833 (2020).
6. Oldham, M. L., Khare, D., Quiocho, F. A., Davidson, A. L. & Chen, J. Crystal structure of a catalytic intermediate of the maltose transporter. *Nature* **450**, 515-521 (2007).
7. Maruyama, Y. et al. Structure of a bacterial ABC transporter involved in the import of an acidic polysaccharide alginate. *Structure* **23**, 1643-1654 (2015).
8. Mirdita, M. et al. ColabFold: making protein folding accessible to all. *Nat. Methods* **19**, 679-682 (2022).
9. Evans, R. et al. Protein complex prediction with AlphaFold-Multimer. Preprint at https://www.biorxiv.org/content/10.1101/2021.10.04.463034v2 (2022).
10. Lee, M. H. & Hatfull, G. F. Mycobacteriophage L5 integrase-mediated site-specific integration *in vitro*. *J. Bacteriol* **175**, 6836-6841 (1993).
11. Andrew, N. et al. Optimisation of bioluminescent reporters for use with mycobacteria. *PLoS One,* **5**, e10777 (2010).
12. Parish, T. & Stoker, N. G. Use of a flexible cassette method to generate a double unmarked *Mycobacterium tuberculosis tlyA plcABC* mutant by gene replacement. *Microbiology* **146**, 1969-1975 (2000).
13. Bardarov, S. et al. Specialized transduction: an efficient method for generating marked and unmarked targeted gene disruptions in *Mycobacterium tuberculosis, M. bovis* BCG and *M. smegmatis*. *Microbiology*, **148**, 3007-3017 (2002).

Uncropped gels and blots

Supplementary Figure 6

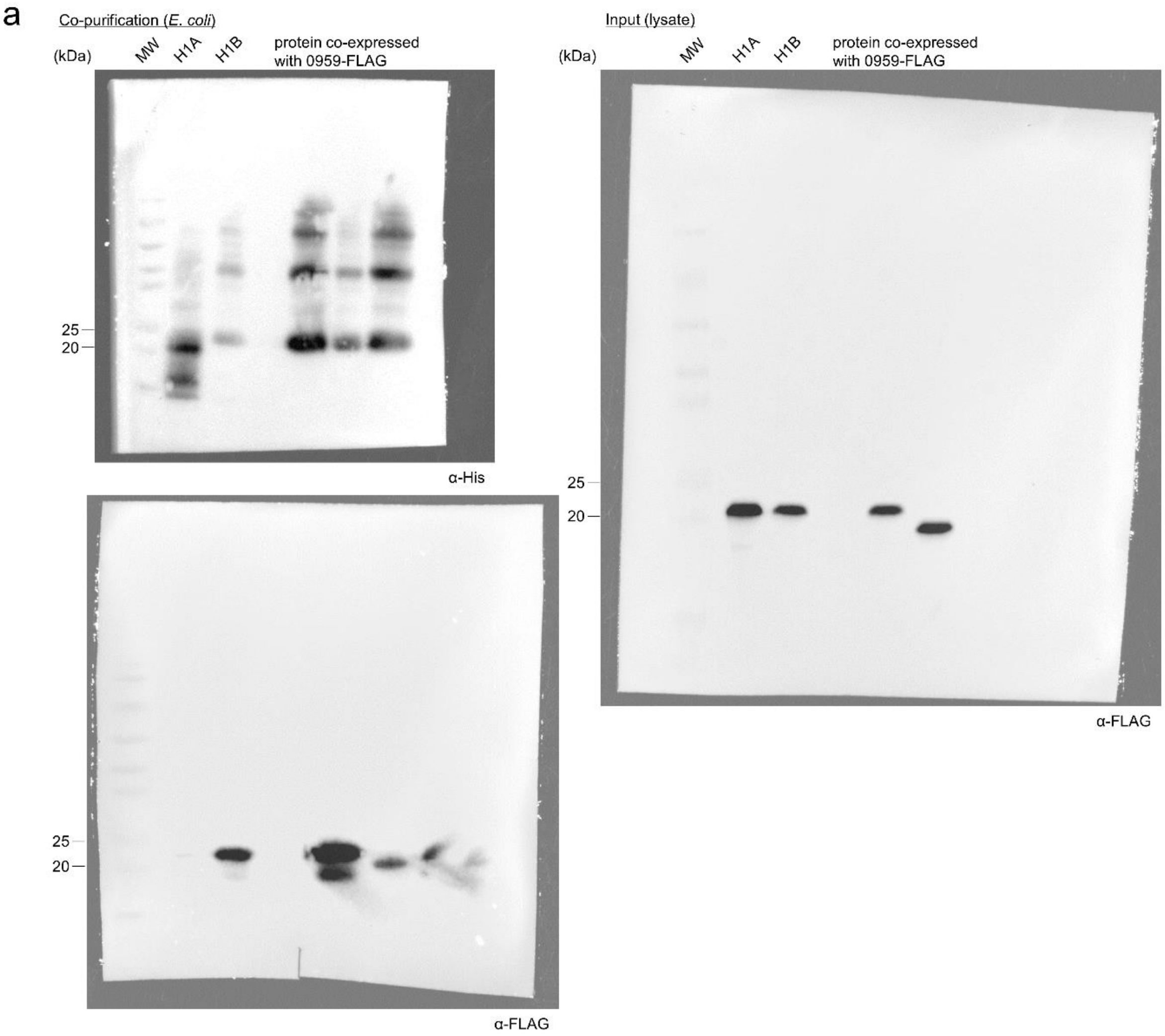

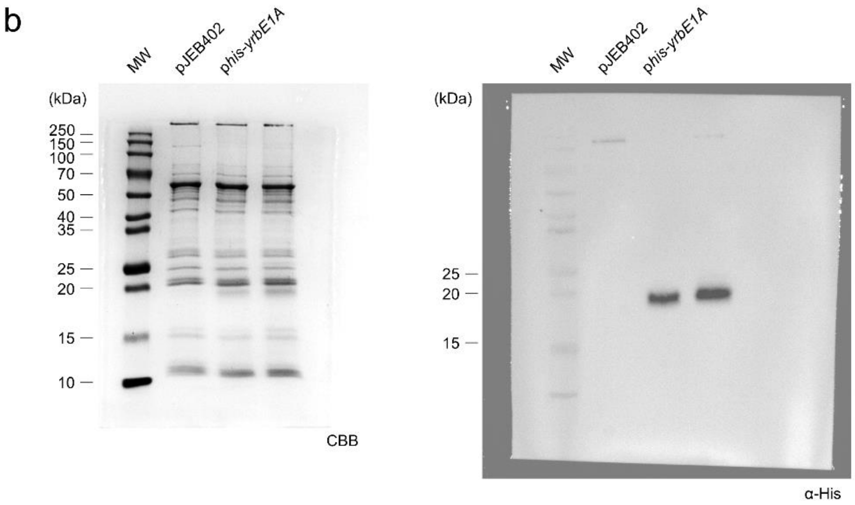

Supplement: Supplementary file 1 — Supplementary Information [file 41467_2023_41578_MOESM1_ESM.pdf]
