# Supplementary material for: A conserved membrane protein negatively regulates Mce1 complexes in mycobacteria
Source: Nat Commun. 2023 Sep 22;14:5897. doi: 10.1038/s41467-023-41578-y (PMC10517005; doi:10.1038/s41467-023-41578-y)
Supplement: Supplementary file 3 — Description of Additional Supplementary Files [file 41467_2023_41578_MOESM3_ESM.pdf]

Description of Additional Supplementary Files

File Name: Supplementary Data 1

Description: AF2 predicted structural models

File Name: Supplementary Data 2

Description: Oligonucleotide sequences
