# Supplementary material for: A conserved membrane protein negatively regulates Mce1 complexes in mycobacteria
Source: Nat Commun. 2023 Sep 22;14:5897. doi: 10.1038/s41467-023-41578-y (PMC10517005; doi:10.1038/s41467-023-41578-y)
Supplement: Supplementary file 5 — Supplementary Data 2 [file 41467_2023_41578_MOESM5_ESM.pdf]

name	sequence (5' to 3')	function
1R5UTRF	AGCTACTAGTGACCTGTTCGGACTCAAGCACATC	to construct the suicide plasmid for *mce1R* deletion by restriction cloning
1R5UTRR	ATATAAGCTTGTGGTCTCCGGTGGTATCTCGGGTG	
1R3UTRF	ACGTAAGCTTCAGATCAGCTCTGGGTGAGC	
1R3UTRR	ATATCCATGGTGCACCGATGCGTTCGAGGACGGCT	
G5UTRF	ATATACTAGTTCGGAAGCCGGCATTGAAGGCCTCGA	to construct the suicide plasmid for *mceG* deletion by restriction cloning
G5UTRR	AGCTAAGCTTCAAAGATCCTTCCCGCTACGCCTACCA	
G3UTRF	AATTAAGCTTGTTTGCCGCGATCAGGCCGGGCCGTCA	
G3UTRR	ATATCCATGGATCCGAGAAGGACAGTGACATCGAG	
1A5UTRF	TGGTAATACGACTCA GAAGGCGACGCTGTTCGGAA	to construct the suicide plasmid for *mce1A* deletion by Gibson assembly
1A5UTRR	TTCTCCC CGCTACACCGTCAGGTTGAAG	
1A3UTRF	GTGTAGCG GGGAGAACACGATCAACCCAT	
1A3UTRF	TATTTCGGACGGTTG CCGTTGAGCTTCAAGGTCA	
65405UTRF	CGTTACTAGTCAAGGCGATGATGGTCTTCTCC	to construct the suicide plasmid for *MSMEG_6540* deletion by restriction cloning
65405UTRR	ATATAAGCTTCGCCGCGGATGAGGCCACCT	
65403UTRF	AGCTAAGCTTCAACCCGTGACTGTCTTGGCT	
65403UTRR	AATTCCATGGCTGCTCGACATCACCGGCA	

1B5UTRF	TGGTAATACGACTCA CCGCCTGGCGTCGATCGACGTCG	to construct the suicide plasmid for *mce1B* deletion by Gibson assembly
1B5UTRR	TGTCCTC ATGGGTTGATCGTGTTCTCCCCCA	
1B3UTRF	CAACCCAT GAGGACACTGCAGGGTTCCGAC	
1B3UTRF	TATTTCGGACGGTTG TCGACGAAGGGCTGCAGGATG	
1C5UTRF	TGGTAATACGACTCA TCGGCATCTTCTCGCTGGTGCT	to construct the suicide plasmid for *mce1C* deletion by Gibson assembly
1C5UTRR	TCCCTGC CTATTTCGGCGTGCACCTACC	
1C3UTRF	CGAAATAG GCAGGGAGGCGTCGAGACATGT	
1C3UTRF	TATTTCGGACGGTTG CCTGGTTGGGCGAGTTGATGTT	
1D5UTRF	TGGTAATACGACTCA AACAAGGTCGCGACGGTGCTCG	to construct the suicide plasmid for *mce1D* deletion by Gibson assembly
1D5UTRR	AGCCTCAT GTCTCGACGCCTCCCTGCCTAT	
1D3UTRF	TCGAGAC ATGAGGCTGCTGAAGGGTTTCC	
1D3UTRF	TATTTCGGACGGTTG AGGTCGAGCTTGAGCGACACGT	
1E5UTRF	TGGTAATACGACTCA GTTTGGCATTGTTCGTCAACGC	to construct the suicide plasmid for *mce1E* deletion by Gibson assembly
1E5UTRR	CCCTTTC TCAGCCTGCTCCTGCTTCAGCGGG	
1E3UTRF	CAGGCTGA GAAAGGGGGGAGTGAGATGCTG	
1E3UTRF	TATTTCGGACGGTTG CACTGCGAGGCGGGCAGGAAGC	
1F5UTRF	ATATACTAGTAGCATGACGCTGTACGTGCAGA	

1F5UTRR	ATATAAGCTTCTCACTCCCCCCTTTCGAC	to construct the suicide plasmid for *mce1F* deletion by restriction cloning
1F3UTRF	ATATAAGCTTAGGAGATGACGGATGGAAGG	
1F3UTRF	ATATCCATGGTCCGGTTACGCGCGTCCCA	
y15UTRF	AATTACTAGTGAAACTGCGTGCGCTGTCGT	to construct the suicide plasmid for *yrbE1A/B* deletion by restriction cloning
y15UTRR	ATATAAGCTTAGGTGCCCTTCCTGGACGTGATC	
y13UTRF	ATATAAGCTTCTGACGGTGTAGCGCCATGAC	
y13UTRR	AATTCCATGGGTACACATACGGGTTGCC	
lZsBF	CGCGGCCGCAATTAACCCTCACTAAAGGATC TTAATTAAAGCCCGCTCATTAGGCGGG	to clone the *lacZ-sacB* cassette using pGOAL17 as the template for insertion into suicide plasmids by Gibson assembly
lZsBR	GGCCGCATAATACGACTCACTATAGGGATC TTAATTAAGCGGCCGCGGTACCAA	
y1AF	ATCACATATGACGGCGTCGACCGATG	to construct pET28b*his*$_{6}$-*yrbE1A* by restriction cloning
y1AR	ATGTAAGCTTTCAGCGCCCTGTCCCGAAC	
y1BF	ATCACATATGAGTACTGTTCAGGTTCTCCGCT	to construct pET28b*his*$_{6}$-*yrbE1B* by restriction cloning
y1BR	ATGTAAGCTTCTACACCGTCAGGTTGAAGTTCG	
opt1NBF	AATTCATATGACTGCGTCTACAGAC	to construct pET22/42*yrbE1A*-(*his*$_{6}$-*yrbE1B*) by restriction cloning with *yrbE1A*-(*his*$_{6}$-*yrbE1B*) cloned from a synthesized *mce1* operon optimized for *E. coli* codon usage
opt1NBR	AGCTAAGCTTCTATACAGTAAGATTAAAGTTAGG	
opt1BF	ACCATGAGTACAGTACAGGTGTTGC	to construct pET22/42*yrbE1A*/*B* by site-directed mutagenesis (SDM) using

opt1BR	TGTACTCATGGTATATCTCCTTCTTAAATC	pET22/42*yrbE1A*-(*his* $_6$-*yrbE1B*) as the template
opt1NAF	CACCATCATCACCACAGCAGCGGCACTGCGTCTACAGACGGGTT	to construct pET22/42(*his* $_6$-*yrbE1A*)-*yrbE1B* by SDM using pET22/42*yrbE1A*/*B* as the template
opt1NAR	GTGATGATGGTGATGGCTGCTGCCCATATGTATATCTCCTTCTTAAAG	
opt1NBGSF	GTGGTAGCAGCGGTGGGGCGATGGCTGTCATC	to construct pET22/42*yrbE1A*-(*his* $_6$-*yrbE1B* $_{GS}$) using pET22/42*yrbE1A*-(*his* $_6$-*yrbE1B*) as the template by SDM
opt1NBGSR	CGCTGCTACCACCCAAAGATTCACGACGGTAGT	
CDFGF	TATACCATGGGCGTCCAAATCGACGT	to construct pCDFDuet1*mceG* by restriction cloning
CDFGR	ATATAAGCTTCACGCCTGCTTGGGCA	
GKAF	ACCGGCGCGTCCGTGTTCCTGAAGTCGCTG	to construct pCDFDuet1*mceG* $_{K43A}$ using pCDFDuet1*mceG* as the template by SDM
GKAR	GGACGCGCCGGTACCGGAGGGGCCCAGCA	
GflF	GACTACAAGGACGATGACGACAAGTGAAGCTTGCGGCCGCATAA	to add a FLAG-tag sequence to *mceG* at the first multiple cloning site of pCDFDuet1 by SDM
GflR	GTCCTTGTAGTCACCGCTGCTACCCGCCTGCTTGGGCACCTCGA	
CDF959F	AATTCATATGACTGAACCCGCAGGCCACGA	to insert *MSMEG_0959* into the second multiple cloning site of pCDFDuet1 by restriction cloning
CDF959R	TATACCTAGGTCACGGCGTGCGCTCTTCCACC	
AGPF	CGGAGCTCACCCCGGTCCCGCGCGGCCGGACC	to convert WT *MSMEG_0959* to *MSMEG_0959* $_{AGG3P}$ on different plasmids by SDM
AGPR	GGGTGAGCTCCGGGAGCGGACGCGCCGACTGCCACTT	
Jy1F	CGGATCCAGCTGCAG GAAGGGCACCTGTGACGG	to construct pJEB402*yrbE1A/B* by Gibson

Jy1R	GTGCGGCCGCGGTAC CTACACCGTCAGGTTGAAGT	assembly
JGF	CGGATCCAGCTGCAG TAAGGAGATATACCATGGGC	to construct pJEB402*mceG* with *mceG* cloned from pCDFDuet1*mceG* as the template by Gibson assembly
JGF	GTGCGGCCGCGGTAC TCACGCCTGCTTGGGCACCT	
J1AF	CGGATCCAGCTGCAG AGGAAGGAGCGCCATGACCGAGCCTCCAGCGC	to construct pJEB402*mce1A* using Gibson assembly
J1AR	ACGTCGACATCGATA CTCATGGGTTGATCGTGTTCT	
J6540F	ATAT<u>GAATTC</u>AGGAGAAGCGCCGTGGCCTCATCCGCGGCGT	to construct pJEB402*MSMEG_6540* by restriction cloning
J6540R	ATAT<u>AAGCTT</u>TCACGGGTTGATGGTGTTCT	
Jm1F	CGGATCCAGCTGCAG AGGAGATGACGGATGGAAGGA	to construct pJEB402*mam1A–D* by Gibson assembly
Jm1R	ACGTCGACATCGATACTACAGCTGCGGCGAGAGGTC	
J1BF	CGGATCCAGCTGCAG AGGAAGGAGACCCATGAGTATCAAAGGCACGCTTT	to construct pJEB402*mce1B* by Gibson assembly; J1BF was also used together with Jm1R to construct pJEB402*mce1B–F*-*mam1A–D*
J1BR	ACGTCGACATCGATA TCATTTCGGCGTGCACCTACC	
J1CF	CGGATCCAGCTGCAG AGGAAGGAGCGAAATGAGGACACTGCAGGGTTCC	to construct pJEB402*mce1C* by Gibson assembly; J1CF was also used together with Jm1R to construct pJEB402*mce1C–F*-*mam1A–D*
J1CR	ACGTCGACATCGATA CTATCTCGACTGCGAACCCGG	
J1DF	CGGATCCAGCTGCAG AGGAAGGAGAGACATGTCAACGATTTTCAACATCC	to construct pJEB402*mce1D* by Gibson assembly; J1DF was also used together with Jm1R to construct pJEB402*mce1D–F*-*mam1A–D*
J1DR	ACGTCGACATCGATA TCAGCCTGCTCCTGCTTCAGC	
J1EF	CGGATCCAGCTGCAG AGGAAGGAGGCTGATGAGGCTGCTGAAGGGTTTCC	to construct pJEB402*mce1E* by Gibson assembly; J1EF was also used together with Jm1R to construct pJEB402*mce1E–F*-*mam1A–D*
J1ER	ACGTCGACATCGATA TCTCACTCCCCCCTTTCGACCAG	

J1FF	CGGATCCAGCTGCAG AAAGGGGGGAGTGAGATGCT	to construct pJEB402*mce1F* by Gibson assembly; J1FF was also used together with Jm1R to construct pJEB402*mce1F*-*mam1A*–*D*
J1FR	GTGCGGCCGCGGTAC TGTGTTCCTGCTGCTGATCG	
pETRBSF	TCGAGAATTCTTAACTTTAAGAAGGAGATATAC	to construct pMV306hsp*his*$_6$-*yrbE1A* and pMV306hsp*his*$_6$-*yrbE1B* by restriction cloning with *his* [illegible]-*yrbE1A* and *his* [illegible]
M959F	ATATGAATTCAGGAGAGACCGTGACTGAACC	to construct pMV306hsp*MSMEG_0959* by restriction cloning
M959R	AATTAAGCTTCACGGCGTGCGCTCTTCC	
959flR	GTCCTTGTAGTCACCGCTGCTACCCGGCGTGCGCTCTTCCACCA	to add a FLAG-tag sequence to *MSMEG_0959* by SDM; used together with M959flF, M959flN1BF or CDF959flF.
M959flF	GACTACAAGGACGATGACGACAAGTGAAGCTTATCGATGTCGACG	to add a FLAG-tag sequence to *MSMEG_0959* on pMV306hsp*MSMEG_0959* by SDM
M959flN1BF	GACTACAAGGACGATGACGACAAGTGACTTAACTTTAAGAAGGAGATATACC	to add a FLAG-tag sequence to *MSMEG_0959* on pMV306hsp*MSMEG_0959+his*$_6$-*yrbE1B* by SDM
CDF959flF	GACTACAAGGACGATGACGACAAGTGACCTAGGCTGCTGCCACC	to add a FLAG-tag sequence to *MSMEG_0959* at the second multiple cloning site of pCDFDuet1*MSMEG_0959* by SDM
MRv0513F	ATATGAATTCAAGGAGATATACATGACACCAACCGGGGATA	to construct pMV306hsp*Rv0513* by restriction cloning
MRv0513R	ATATAAGCTTCGGCACAAGCCAGGAACAGTT	
MRv0513N1BF	GGGATCCAGCTGCAG AAGGAGATATACATGACACCAACCGGGGATA	to construct pMV306hsp*Rv0513* +*his*$_6$-*yrbE1B* by Gibson assembly
MRv0513N1BR	ATCTCCTTCTTAAAGTTAAG CGGCACAAGCCAGGAACAGTT	

Rv0513flF	GACTACAAGGACGATGACGACAAGTGATCGCCCGCTACCGG	to add a FLAG-tag sequence to *Rv0513* on pMV306hsp*Rv0513+his* $_6$*-yrbE1B* by SDM
Rv0513flR	GTCCTTGTAGTCACCGCTGCTACCCCGCGCTGGCCCGGCGT	
959L55CF	GATCTGCCAGCGCACCGGCGGTTAC	to introduce a L55C mutation to MSMEG_0959 by SDM
959L55CR	CGCTGGCAGATCGCCATGCCGACACC	
959G59CF	CACCTGCGGTTACGGCTATGACCTG	to introduce a G59C mutation to MSMEG_0959 by SDM
959G59CR	TAACCGCAGGTGCGCTGCAGGATCGC	
959G60CF	CGGCTGCTACGGCTATGACCTGTGG	to introduce a G60C mutation to MSMEG_0959 by SDM
959G60CR	CCGTAGCAGCCGGTGCGCTGCAGGAT	
959Y61CF	CGGTTGCGGCTATGACCTGTGGATC	to introduce a Y61C mutation to MSMEG_0959 by SDM
959Y61CR	TAGCCGCAACCGCCGGTGCGCTGCAG	
1BE208CF	GTACTGCCACTACTTCCACACGTTC	to introduce a E208C mutation to YrbE1B by SDM
1BE208CR	TAGTGGCAGTACGTGCCGACCGACTG	
1BF211CF	CTACTGCCACACGTTCCTGCGTGTC	to introduce a F211C mutation to YrbE1B by SDM
1BF211CR	GTGTGGCAGTAGTGCTCGTACGTGCC	
1BH212CF	CTTCTGCACGTTCCTGCGTGTCGAC	to introduce a H212C mutation to YrbE1B by SDM
1BH212CR	AACGTGCAGAAGTAGTGCTCGTACGT	

Mycobacterium smegmatis mc2 155 genomic DNA was used as the template for cloning unless stated otherwise. Restriction sites are underlined. A space is left between the sequence annealed to the template and the sequence used as an overhang for Gibson assembly. SDM, site-directed mutagensis.
