# Supplementary figures and images for: A conserved membrane protein negatively regulates Mce1 complexes in mycobacteria
Source: Nat Commun. 2023 Sep 22;14:5897. doi: 10.1038/s41467-023-41578-y (PMC10517005; doi:10.1038/s41467-023-41578-y)

Figure 1c


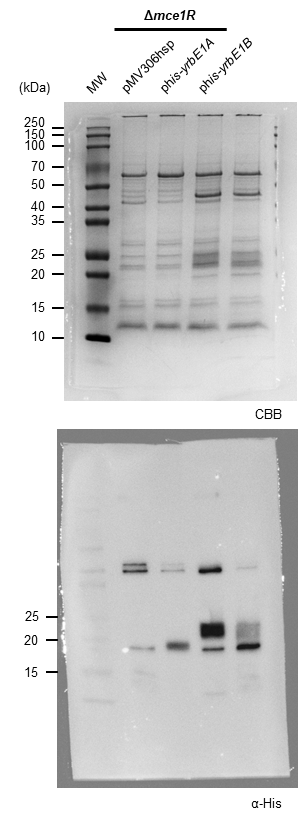


Figure 3a inset


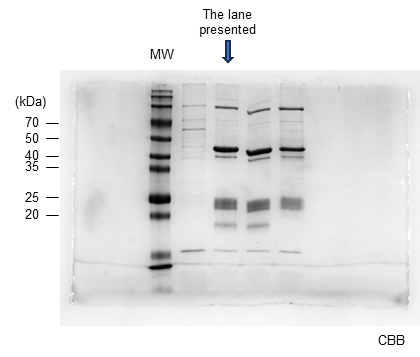


Figure 3c


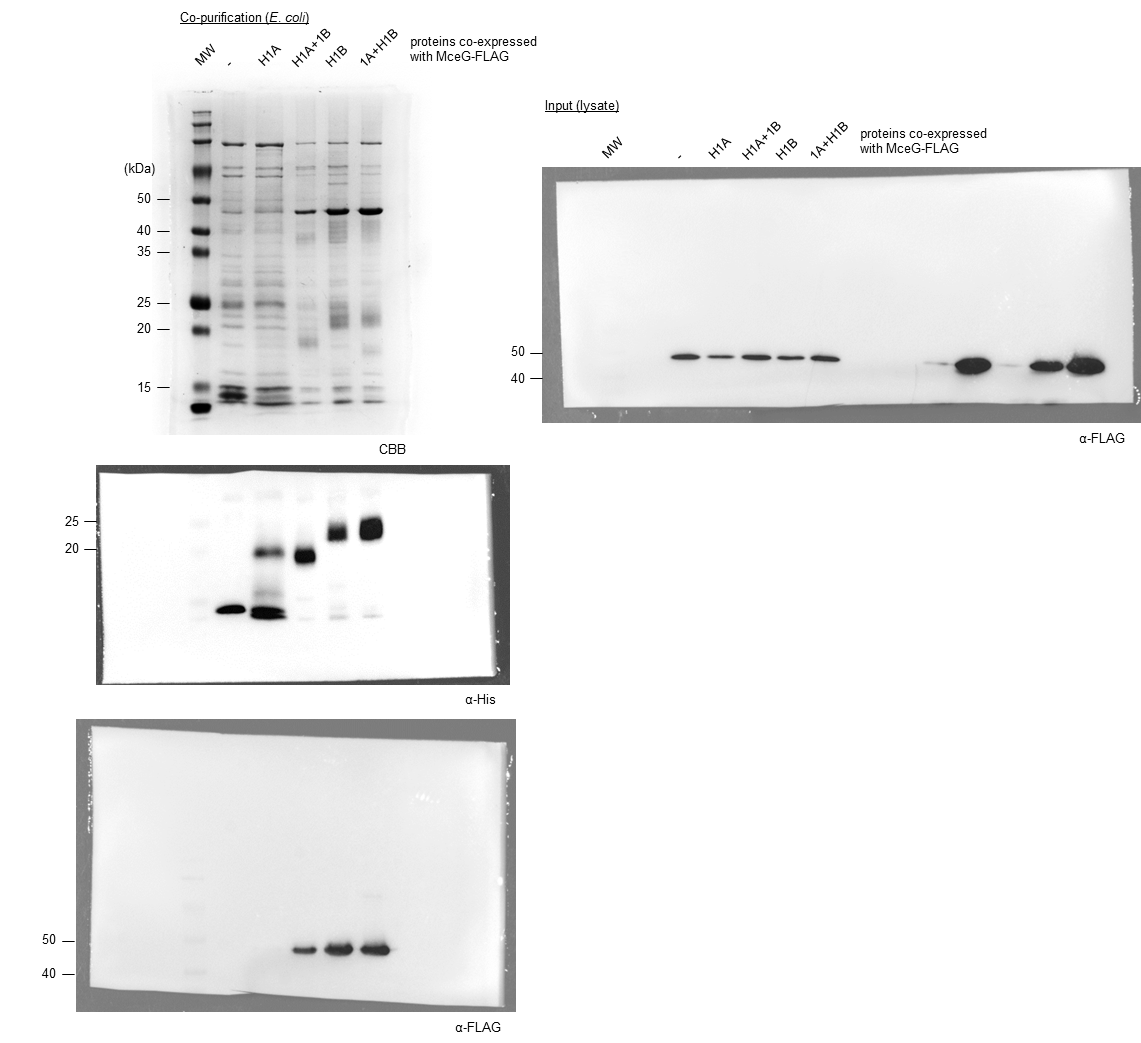


Figure 4a


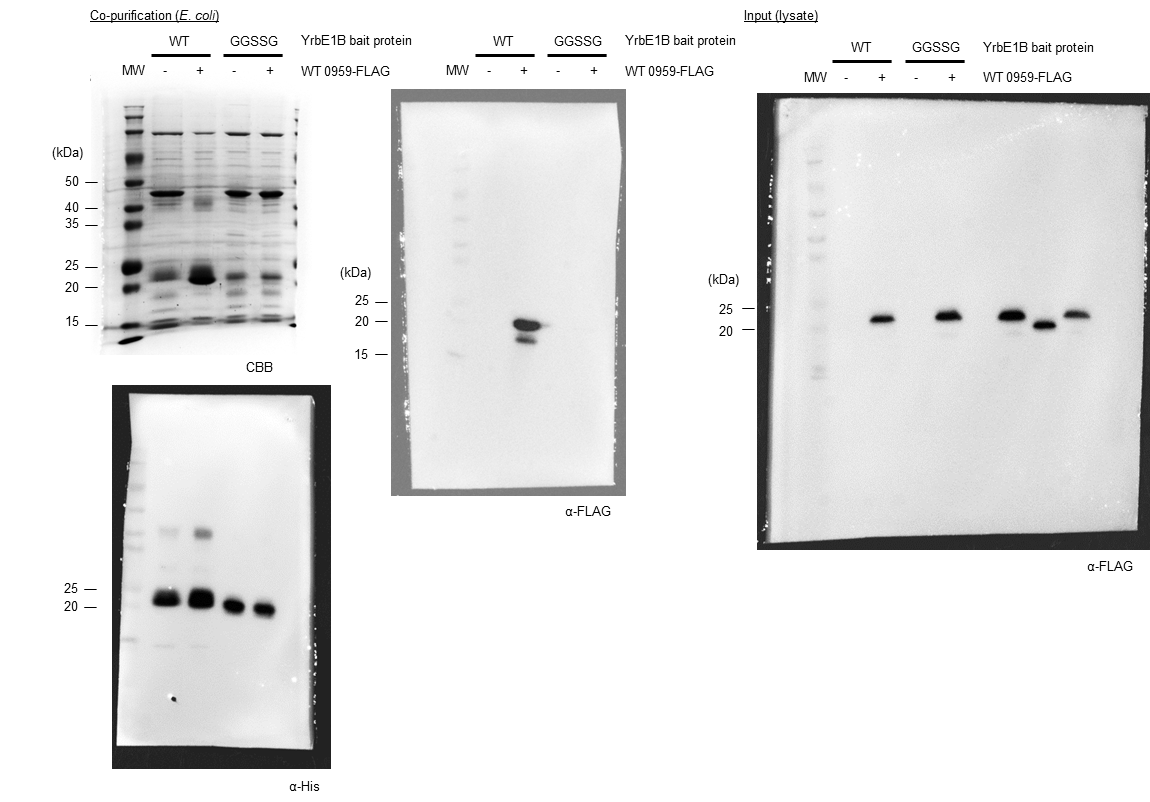


Figure 4b


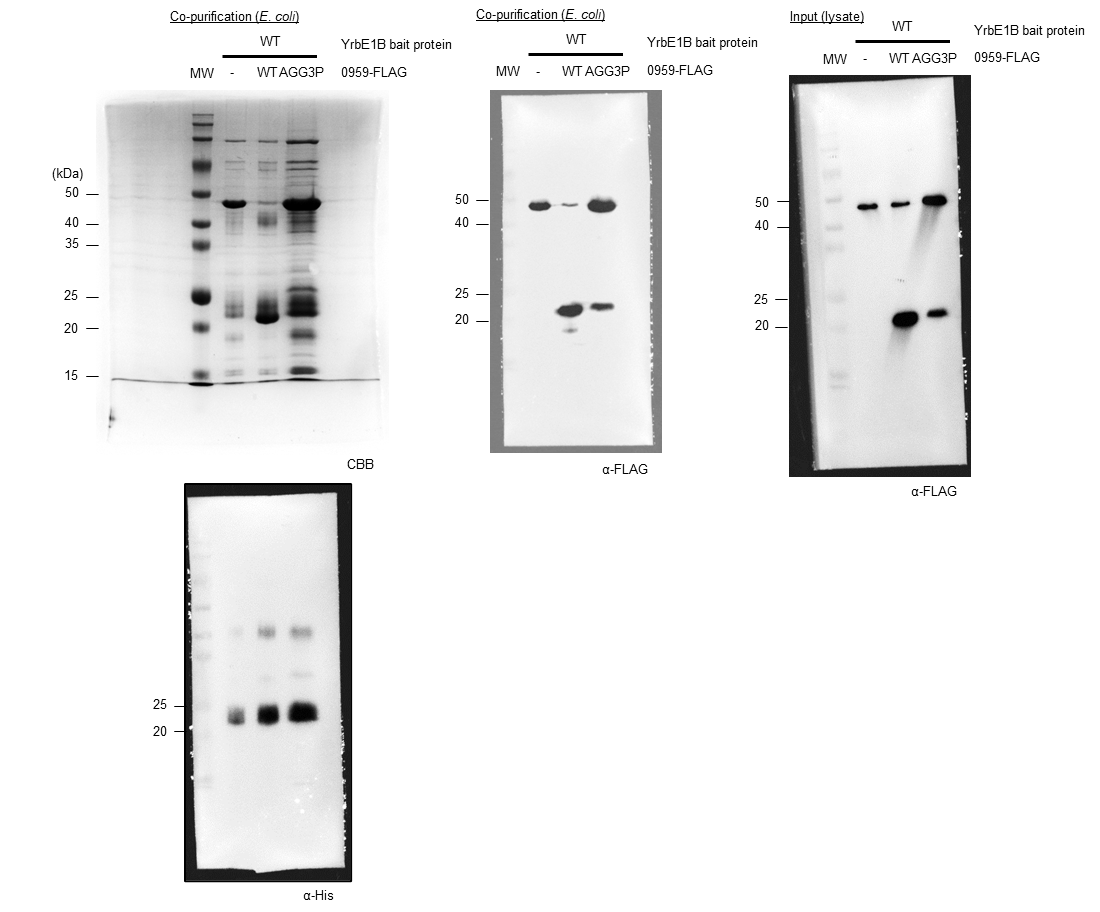


Figure 4c


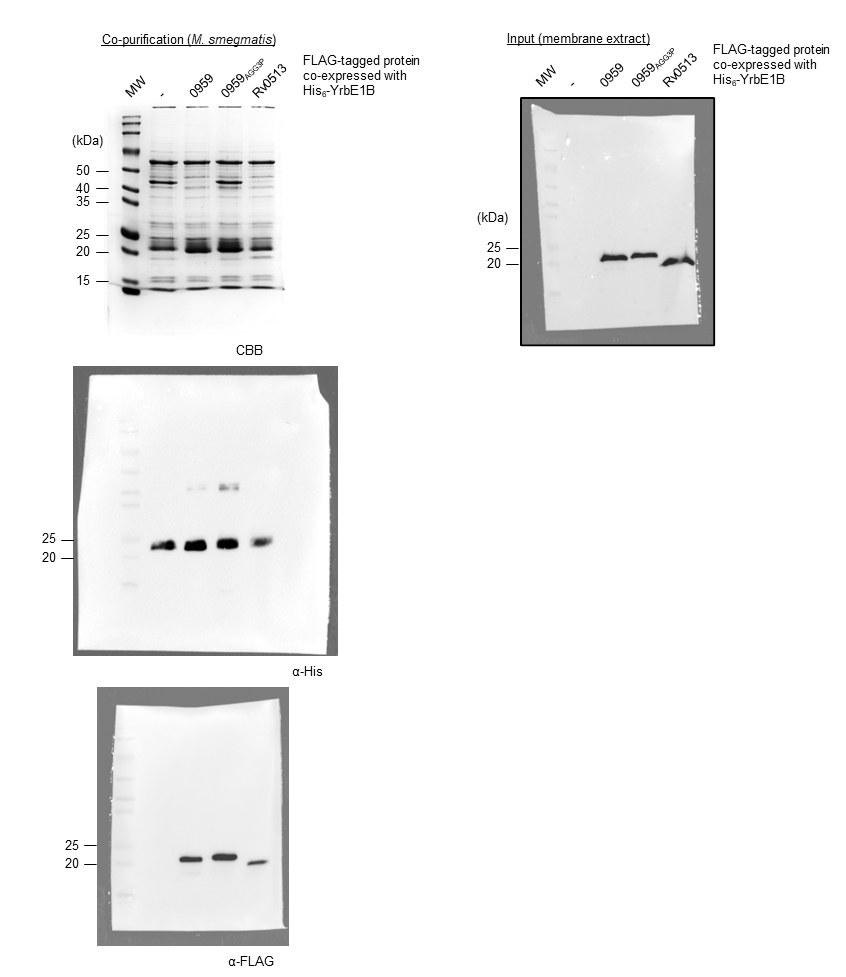


Figure 5b


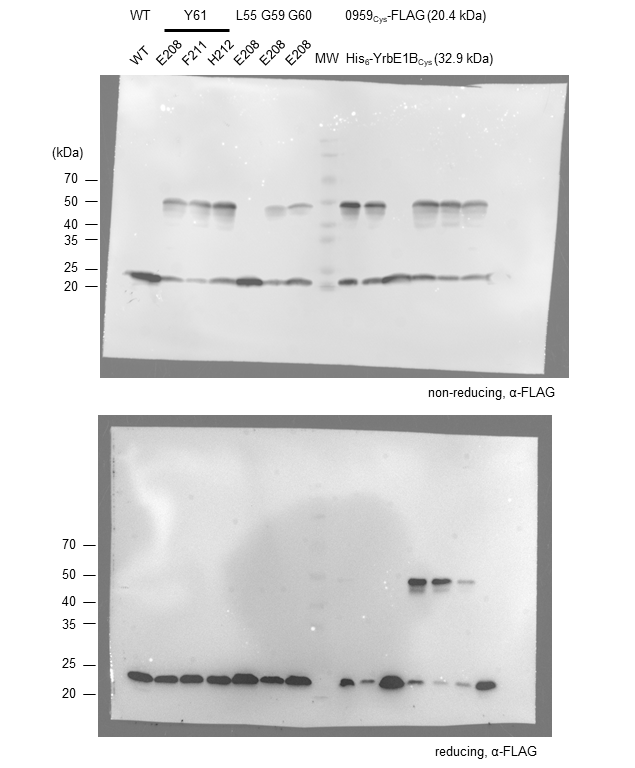


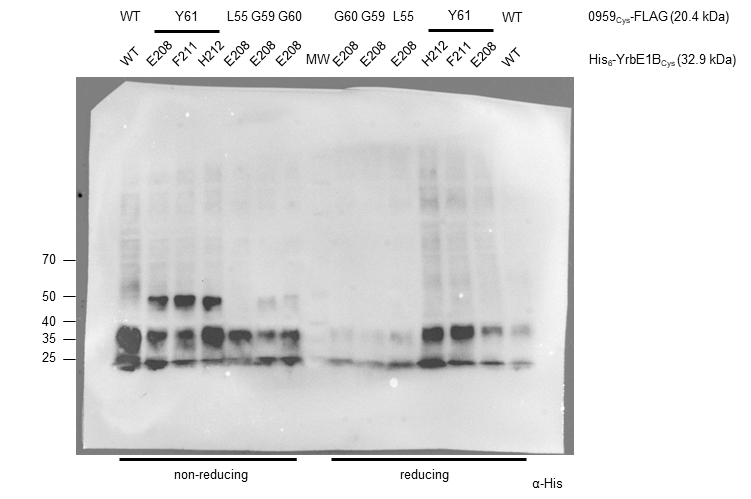

Supplement: Supplementary file 7 — Source Data [file 41467_2023_41578_MOESM7_ESM.zip › Source Data uncropped gels and blots.docx]
